# Medical specialists’ basic psychological needs, and motivation for work and lifelong learning: a two-step factor score path analysis

**DOI:** 10.1186/s12909-019-1754-0

**Published:** 2019-09-05

**Authors:** Stéphanie M. E. van der Burgt, Rashmi A. Kusurkar, Janneke A. Wilschut, Sharon L. N. M. Tjin A Tsoi, Gerda Croiset, Saskia M. Peerdeman

**Affiliations:** 1 Amsterdam UMC, Vrije Universiteit Amsterdam, Research in Education, VUmc School of Medical Sciences, de Boelelaan 1117, Amsterdam, The Netherlands; 2 Department of Epidemiology & Biostatistics, VU University Medical Center Amsterdam, Amsterdam, The Netherlands; 3 PAOFarmacie, The Netherlands Centre for Post-Academic Education in Pharmacy, Amsterdam, The Netherlands; 4 Department of Neurosurgery, VU University Medical Center Amsterdam, Amsterdam, The Netherlands; 5 LEARN! Research Institute for Learning and Education, Faculty of Psychology and Education, VU University Amsterdam, Amsterdam, The Netherlands

**Keywords:** Medical specialists, Motivation, Self determination theory, Two step factor path analysis

## Abstract

**Background:**

Continuing professional development and lifelong learning are crucial to secure safe and good quality healthcare. Lack of motivation has been found to be among the most important barriers for participation in lifelong learning. This study was conducted to investigate the relationships between medical specialists’ work motivation, lifelong learning motivation, autonomy, competence and relatedness satisfaction.

**Methods:**

Self-Determination Theory was used as a theoretical framework for this study. Data were collected through an online survey, that was sent to all (*N* = 1591) medical specialists in four Dutch hospitals. The survey measured background characteristics, autonomy, competence, and relatedness satisfaction, autonomous and controlled work motivation, and lifelong learning motivation. Two step factor path analysis with the method of Croon was used to analyze the data from 193 cases.

**Results:**

Autonomy need satisfaction was positively associated with autonomous work motivation which in turn was positively associated with lifelong learning motivation. Competence need satisfaction and age were negatively associated with controlled work motivation. Competence need satisfaction was also positively related with lifelong learning motivation. No significant nor any hypothesized associations were found for relatedness.

**Conclusions:**

Our findings, in line with Self-determination Theory literature, show that autonomy and competence need satisfaction are the important factors as they were positively associated with medical specialists’ motivation for work and for lifelong learning.

## Background

A recent study reported 970 preventable adverse events in Dutch hospitals per year [1]. Globally, the rates of poor performance (which is measured by preventable adverse events) vary from 0.5 to 12% [2]. These poor performance rates lead to reduced quality of care and patient safety. Through professional development, i.e., lifelong learning, medical specialists maintain their professional competence and are able to keep track of and respond to advancing knowledge in their field [3, 4, 9–12]. Continuing professional development (CPD) and lifelong learning as part of CPD are crucial to secure high quality healthcare, patient safety, and societal trust in the healthcare system [3–5]. While learning and development opportunities are energizing factors for practicing healthcare professionals, lack of motivation, time, and funding constitute the most significant barriers for participation in CPD and lifelong learning [5–8]. Since lack of motivation is mentioned as a barrier, an insight into the motivation mechanism of medical specialists would be useful as this makes it possible to enable the development of an optimal environment for specialists to work in, as well as to keep medical specialists participating in lifelong learning. However, to our knowledge, little is known about the motivation of medical specialists for medical practice (work motivation) and for lifelong learning/CPD in particular. Accordingly, the focus of this study is on the work motivation of medical practice and lifelong learning of medical specialists.

**Fig. 1:**
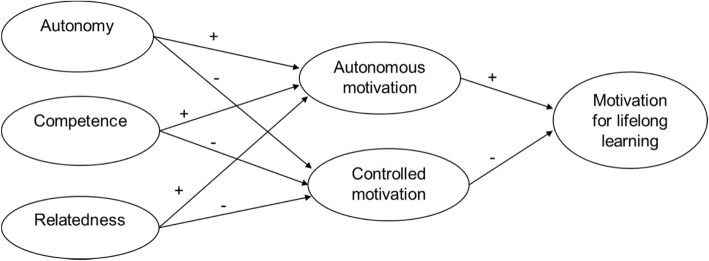
Hypothesized model with all latent variables

The aim of this study is to investigate the score on autonomous motivation (AM), controlled motivation (CM) and lifelong learning motivation as well as the relationship between work motivation (AM and CM), motivation for lifelong learning and the satisfaction of the three basic psychological needs: autonomy, competence and relatedness. The proposed model for this study is illustrated in Fig. 1. The three basic needs and autonomous and controlled motivation are not considered to be independent. Thus, they are often found to be related. The hypotheses that were specified based on the literature are as follows:

1. Autonomy, competence, and relatedness are positively associated with medical specialists’ AM for work and lifelong learning motivation and negatively associated with medical specialists’ CM for work.
2. AM for work is positively associated with medical specialists’ lifelong learning motivation.
3. CM for work is negatively associated with medical specialists’ lifelong learning motivation.

We used the Self-determination theory (SDT) of Deci and Ryan as the framework for the current study [13–17]. SDT classifies motivation on a dynamic continuum and emphasizes the importance of the quality of the motivation. This sets it apart from other theories which put more emphasis on the quantity of the motivation. Several different states of quality of motivation are aligned along the continuum: amotivated, external regulation, introjected regulation, identified regulation, integrated regulation, and intrinsic motivation. Of these, external and introjected regulation together form CM. Identified and integrated regulation and intrinsic motivation together form AM. According to SDT AM comes from within the individual out of interest for and value of the task itself, and is a facilitator and stimulator of deep-level learning and academic performance; it also leads to improved wellbeing, resilience, and patient safety [13–17]. CM is feeling pressured or coerced by external factors or from within and is associated with less desirable outcomes like procrastination and surface-level learning [13–17].

Additionally, within SDT, three basic psychological needs are distinguished: perceived autonomy (experiencing behavior as choiceful and self-endorsed), perceived competence (experiencing behavior as masterful), and relatedness (feeling mutually connected with peers and important others) [13–17]. Perceived competence in this study is the person’s perception of their own competence, rather than professional competence which is generally measured by others such as supervisors or peers. When these basic needs are satisfied this promotes a person’s psychological growth, healthy functioning, and AM. When these needs are frustrated or thwarted this contributes to malfunction, reduced energy and wellbeing, and CM [14, 19–21].

## Methods

### Setting and sample

A quantitative study was conducted in an academic hospital (VU University Medical Center in Amsterdam), a large merged medical center (Noordwestgroep Alkmaar and OLVG Amsterdam), and two affiliated hospitals (Westfriesgasthuis Hoorn and Rode Kruis Ziekenhuis Beverwijk). An online questionnaire was sent to all the medical specialists working in these hospitals. In this study, our definition of a medical specialist is a physician who has completed specialty training.

### Data collection

The online questionnaire included standardized validated scales measuring work motivation (with subscales for AM and CM), the motivation for lifelong learning, and the basic psychological needs of autonomy, competence and relatedness. Additionally, we included the following questions about the background characteristics: sex, age, type of specialty, type of hospital that they work in, and number of years of experience as a medical specialist. The validated scales were translated from English into Dutch by two researchers. They were then back translated by two other researchers to ensure the appropriate Dutch translation [22].

### Measures

The 19-item multidimensional work motivation scale (MWMS) [23] was used to measure the work motivation of the medical specialists. This scale could be divided into AM and CM for work. The stem of the scale is “Why do you or would you put efforts into your current job?” with items like; e.g., “Because I personally consider it important to put efforts into this job.” Responses were made on a seven point Likert scale. All responses to the questions were added together, with the higher scores indicating a higher level of motivation. For this scale Cronbach’s alpha was 0.83 for AM and 0.79 for CM.

The 14-item revised Jefferson Scale of Physician Lifelong Learning (JeffSPLL) [24] was used to measure the medical specialists’ motivation for lifelong learning. The stem of the scale is “Please indicate the extent of your agreement with each of the following statements by circling the appropriate number” with items like: “I believe that I would fall behind if I stopped learning about new developments in my profession.” Responses were made on a four-point Likert scale and were also added together. Here, higher scores also indicate a greater orientation and motivation toward lifelong learning. For this scale Cronbach’s alpha was 0.85.

The Basic Psychological Needs at Work Scale (BPNWS) [25] assessed the perceived autonomy and competence at work of medical specialists. Both subcategories included eight items, e.g.: “I feel my choices in my job express who I really am” (for autonomy) and “When I am at work, I feel competent to achieve my goals” (for competence). Responses were made on a seven point Likert scale. The Cronbach’s alpha was 0.71 for autonomy and 0.78 for competence. Relatedness of medical specialists toward their colleagues was measured with the TEAM Climate Inventory scale (TCI) [26], which included 12 items, e.g.: “People feel understood and accepted by each other.” Responses were made on a five point Likert scale. For this scale, the Cronbach’s alpha was 0.92.

### Analysis

Descriptive statistics were used to assess demographic data such as gender and years of experience. Data were checked for normality distribution and the assumption of a normal multivariate distribution was met. In order to investigate reliability and to get information on validity we computed Cronbach’s alphas. Pearson’s correlations of all variables were also computed. These analyses were performed using the SPSS 22.0 software program. We then performed a factor score path analysis with Mplus 7.0, using the method of Croon [27–30], to investigate the hypothesized association shown in Fig. 1. To overcome sample size issues, the two step factor score regression (FSR) approach is often used instead of SEM analysis. In this approach the first step is to perform a factor analysis and to calculate factor scores for each latent variable. These factor scores are estimates for the true latent variable scores. In the second step, the factor scores are used in a linear regression, as if they were the true latent variable scores. However, the use of factor scores results in biased estimates of the regression parameters. Croon developed a FSR method that corrects for this bias by using an estimation of the variances and covariances of the true latent variable scores instead of the factor scores [27–29]. This is the method that we have used. Additionally, because our hypothesized model includes mediational relationships we performed a series of linear regression analyses/path analysis. The method can be summarized as follows:

1. Perform factor analysis for all latent variables separately and calculate their respective factor scores.
2. Calculate the variance-covariance matrix of the factor scores.
3. Estimate the true variances and covariances for all elements in this variance-covariance matrix.
4. Perform a path analysis using estimated variances and covariances as the input covariance matrix for the model.

Modelfit was assessed using the following criteria: a chi-square, a *p*-value of > 0.05, a comparative fit index (CFI) of > 0.95, a Tucker Lewis index (TCI) > 0.95 and a root mean square error approximation (RMSEA) of < 0.06.

## Results

Out of 1591 medical specialists, a total of 193 specialists from 30 different specialties completed our questionnaire, resulting in a response rate of 12.1%. According to the power analysis that we conducted we needed a minimum of 180 cases. The a priori power analysis was conducted using two tailed tests with a medium effect size of 0.3, an alpha error probability of 0.05 and a power of 0.95.

Of the specialists, 85 (43.8%) were male and 108 (56.2%) were female, the mean age was 49 years, and 56.2% of the specialists worked in a non-academic hospital. Table 1 reports the participants’ mean scores on the different types of work motivation and the basic psychological needs satisfaction. Table 1 also shows the division of the specialties into three groups; surgical, non-surgical and supportive, which are based on the division that is used by NIVEL, the National institute for health research in the Netherlands [31]. NIVEL uses a division of six groups: First-line curative care (i.e. general practitioner), Public healthcare (i.e. occupational physician), Psychiatry (except psychiatrist working at a hospital), Surgical (all specialties that work in the operating theatre), Non-surgical (i.e. dermatologist, cardiologist) and Supportive (i.e. anesthesiologist, pathologist). However, the groups first-line care, public healthcare and psychiatry were not applicable to our study as these specialists are not working in hospitals. Missing data was handled per variable because of the already small sample size. Some variables did not have any missing data and therefore have the complete number of 193 participants with 183 as smallest N on the variable type of hospital. Other variables did have missing data and therefore have a total number of participants that is lower than 193. Differences between mean scores were tested for significance by using a t-test. For differences between type of specialty ANOVA was used.

**Table 1:** Mean scores on AM (autonomous work motivation), CM (controlled work motivation), lifelong learning motivation, and basic psychological need satisfaction

	N(%)	AM	CM	Lifelong learning motivation	Autonomy satisfaction	Competence satisfaction	Relatedness satisfaction
Gender							
Male	84 (43.5)	5.66	3.28	3.21	4.34	3.85	3.84
Female	108 (56.5)	5.87	3.35	3.11	4.40	3.82	3.67
		ns	ns	ns	ns	ns	ns
Age							
*< 50 years*	106 (54.9)	5.89	3.41	3.11	4.36	3.82	3.79
*> 50 years*	87 (45.1)	5.66	3.20	3.21	4.38	3.84	3.72
		*p* < 0.05	ns	ns	ns	ns	ns
Years of experience							
*< 15 years*	110 (57)	5.84	3.43	3.10	4.36	3.83	3.75
*> 15 years*	83 (43)	5.71	3.17	3.22	4.37	3.87	3.76
		ns	*p* < 0.05	*p* < 0.05	ns	ns	ns
Type of hospital^a^							
Academic	75 (38.9)	5.84	3.30	3.23	4.45	3.89	3.64
Non- academic	108 (56.1)	5.81	3.38	3.09	4.43	3.80	3.82
		ns	ns	*p* < 0.05	ns	ns	ns
Type of Specialty^a^							
Surgical	49 (25.9)	5.89	3.21	3.17	4.26	3.82	3.90
Non-surgical	95 (50.3)	5.76	3.39	3.15	4.38	3.83	3.69
Supportive	45 (23.8)	5.68	3.22	3.13	4.43	3.78	3.74
		ns	ns	ns	ns	ns	ns

Mean scores of AM and CM are based on a seven point Likert scale, lifelong learning motivation on a four point Likert scale and basic psychological needs on a five point Likert scale.

^a^Less than 193 cases as not all participants answered the questions

Table 1: Mean scores on AM (autonomous work motivation), CM (controlled work motivation), lifelong learning motivation, and basic psychological need satisfaction.

Before conducting the factor score path analysis, Pearson correlations were calculated (Table 2). Three significant Pearson correlations were found. AM and motivation for lifelong learning were significantly positively correlated. Autonomy and competence need satisfaction were both significantly positively correlated with CM.

**Table 2:** Pearson correlations of autonomous, controlled and lifelong learning motivation; and autonomy, competence and relatedness satisfaction

	AM (Autonomous work motivation	CM (controlled work motivation)	Lifelong learning motivation	Autonomy satisfaction	Competence satisfaction	Relatedness satisfaction
AM (Autonomous Work Motivation)	1					
CM (Controlled Work Motivation)	−0.005	1				
lifelong learning motivation	0.342*	−0.042	1			
Autonomy satisfaction	0.107	0.154*	0.012	1		
Competence satisfaction	0.115	0.220*	0.125	0.109	1	
Relatedness satisfaction	0.135	−0.027	0.057	−0.051	0.070	1

*p < 0.05

Following the four steps of the FSR with the Croon method we first performed a factor analysis for all latent variables and calculated their factor scores. Factor scores were calculated using the regression predictor. Factor loadings are presented in the Table 4 in Appendix, followed by Table 5 in Appendix that shows the goodness of fit for all variables. Because all scales have been validated thoroughly before and the Cronbach’s alphas were all quite high the model fit for all variables was good. Secondly we calculated the variance-covariance matrix for all factor scores. For the third step we estimated the true variances and covariances for all elements in the variance-covariance matrix. The results are shown in Table 3.

**Table 3:** True variances and covariances for all elements

	AM (Autonomous Motivation)	CM (Controlled motivation)	Lifelong learning motivation	Autonomy satisfaction	Competence satisfaction	Relatedness satisfaction
AM (Autonomous Motivation)	0.559					
CM (Controlled motivation)	− 0.003	0.665				
Lifelong learning motivation	0.108	−0.012	0.132			
Autonomy satisfaction	0.035	0.059	0.005	0.224		
Competence satisfaction	0.032	0.067	0.018	0.020	0.142	
Relatedness satisfaction	0.052	−0.016	0.020	−0.012	0.016	0.405

**Fig. 2:**
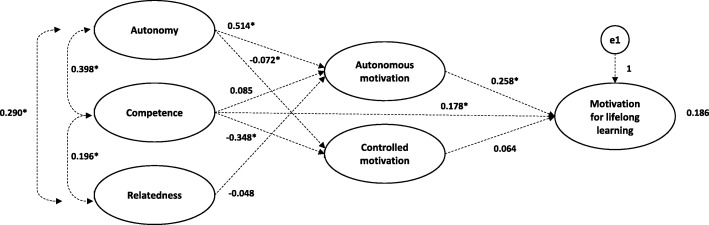
Final model of the structural relations identified. *p < 0.05

As a final step we performed a path analysis using estimated variances and covariances as the input covariance matrix for the model. This provided us the following fit indices of our hypothesized model: X^2^ = 0.463 (df = 4, *p* = 0.977), CFI = 1, TFI = 1, and RMSEA = 0.00. The CFI and TFI being 1 shows that this model is overfitting, it is more complex than it should be. Therefore, we re-specified and assessed the model based on statistical output and theoretical relevance. Figure 2 depicts our final model with a good model fit following from the fit indices: X^2^ = 23.681 (df = 16, *p* = 0.097), CFI = 0.950, and RMSEA = 0.05.

Perceived autonomy was positively associated with AM for work which in turn was positively associated with lifelong learning motivation (Fig. 2). Competence was negatively associated with CM for work. Competence was also directly positively associated with lifelong learning motivation. Experiencing autonomy and competence contributes to medical specialists being more motivated for lifelong learning. No significant relationship was found for relatedness.

## Discussion

The aim of this study was to investigate the relationship between work motivation (AM and CM), motivation for lifelong learning, and the three basic psychological needs of Self-determination Theory.

We expected autonomy, competence, and relatedness satisfaction to be positively associated with medical specialists’ AM for work and motivation for lifelong learning, and to be negatively associated with medical specialists’ CM for work. We did observe a positive association between autonomy satisfaction and AM and a negative association between competence satisfaction and CM. Furthermore, competence satisfaction had a direct significant positive association with specialists’ motivation for lifelong learning. However, no significant associations for relatedness satisfaction were found, which could suggest that relatedness is difficult to measure quantitatively. Another explanation could be that autonomy and competence are more important than relatedness for work and lifelong learning motivation. A third explanation can be that relatedness was measured with the TCI scale and the other two basic needs were measured with BNWS.

Second, we expected AM for work to be positively associated with medical specialists’ lifelong learning motivation. This is indeed the case in the present study, thus in line with SDT [13–15, 18]. When a specialist has a higher AM for work, it stimulates their daily task-related motivation. One such task is CPD, i.e., lifelong learning. If a medical specialist likes their work, they are more likely to continue learning about it. AM also functions as a mediator for the association of autonomy with the motivation for lifelong learning.

Third, the expectation was that CM for work would be negatively associated with medical specialists’ motivation for lifelong learning. However, no significant association between CM and lifelong learning motivation was found. Although this is not in line with SDT, another study on motivation for lifelong learning among pharmacists had the same results [32]. One potential explanation is that other predictors (autonomy, competence and AM) are so strongly associated with the motivation for lifelong learning that CM for work has no significant role. The possibility of the basic psychological needs satisfaction as predictors for learning outcomes (in this case, lifelong learning) is supported by earlier studies that were conducted in different context with workers, nurses, pharmac\ists, and across different cultures within SDT [21, 32, 33].

We noted a few significant findings from the results of the background characteristics on the different types of motivation. The type of hospital had a significant negative association with the motivation for lifelong learning. This indicates that working in a non-academic hospital is connected to a lower motivation for lifelong learning than working in an academic hospital. One explanation might be that the combination of patient care, research, and education in an academic setting challenges medical specialists’ knowledge and competence in a more autonomous way. It is also possible that working in a non-academic hospital obligates medical specialists to spend time on production and funding, which takes away time and joy from other tasks/factors like patient care, which actually enhance AM. However, medical specialists who choose to work in an academic hospital could already be more autonomously motivated. Medical specialists with more years of experience score lower on CM for work. These results are also in line with research that showed that pharmacists working for more than 10 years are found in more autonomous motivation profiles [32]. Volkening et al. demonstrated that AM significantly increased with age [34]; however, older medical specialists do not score higher on AM for work. One explanation could be that as medical specialists gain more experience, the interventions become more routine and less challenging. This could take away from feeling competent and in turn from being autonomously motivated.

Autonomy and competence satisfaction seem to be the most important for basic needs for lifelong learning motivation of medical specialists. However, autonomy is currently being significantly thwarted in healthcare systems. Rules and regulations are becoming increasingly dominant in healthcare and are likely to decrease autonomy among specialists and make specialists feel more like administrative employees than physicians [36]. Because of the continuous and rapid technological and social developments, there could be a reduction in experienced competence and therefore in AM for work and lifelong learning motivation.

To support medical specialists’ participation in lifelong learning, measures need to be taken to reinstate their sense of autonomy and competence. When work contexts support the basic needs satisfaction this is perceived to not only stimulate optimal motivation, functioning, and wellbeing among employees, but also has benefits for the organization [37]. This could include empowering specialists to be autonomous in their time planning, develop a customized professional development route, and provide for the specialists to devote most of their time to patient care and teaching, from which they derive most of their inspiration [35, 36]. The findings suggest more tailored lifelong learning pathways where specialists can decide themselves whether to and how to fulfill their individual motivational needs for AM for work and lifelong learning. Diverse options for learning formats can be offered, such as hands-on courses, e-learning, workshops and so on, which would enable specialists to make choices in their learning.

### Limitations and future research

The work motivation scale we used has been validated in many professions; however, it has not been used among healthcare professionals. Thus, further validation of this scale among healthcare professionals is necessary. While the sample in this study is small and the response rate low we had sufficient power to detect significant differences. It is common knowledge that medical specialists are sent an overwhelming amount of questionnaires, and they already have too little time to do their daily job. Thus, it is possible that it takes motivated specialists to participate in the questionnaires in the first place. If this is the case, then it might be that the level of motivation can be overestimated in this study. Considering the context, the response rate is reasonable for this population and above the minimum number according to the power analysis.

Moreover, the questionnaires we used consisted of self-assessment scales. Respondents tend to overestimate themselves when filling out these scales. This could mean that the results provided an overestimation of the level of motivation. If this is indeed the case, the need to reinstate medical specialists’ perceived autonomy and competence is even more urgent.

For the measurement of years of experience, we assumed that specialists gain competence as they build their experience (measured by longevity). It seems logical to assume that there is a positive correlation between competence and experience. However, in many settings, especially those where physicians take on non-clinical responsibilities, such as teaching, administration, and research, the level of (patient) experience is reduced by these other activities. For future research, other measures of experience need to be considered, such as the number of procedures conducted or the number of patients seen, rather than longevity.

Although SDT is a universal theory that has been validated in many life domains and across cultures, not much is known about the motivation of medical specialists. More research in other healthcare contexts is necessary to determine the generalizability of our findings. Future research on relatedness among medical specialists, mainly qualitative, is needed to determine how this basic need can be fulfilled. Moreover, measuring relatedness with a different scale (TCI) than the one for other basic needs might be a limitation of this study.

## Conclusion

Our findings, in line with the SDT literature, show that autonomy and competence satisfaction are the most important factors for medical specialists’ motivation for work and lifelong learning. These factors should be taken into account when designing interventions to optimize specialists’ motivation.

## Data Availability

The dataset generated and analyzed during the current study is available from the corresponding author on request.

## Appendix

**Table 4:** CFA factor loadings on all variables

Item	Autonomous motivation	Controlled motivation	Lifelong learning motivation	Autonomy satisfaction	Competence satisfaction	Relatedness satisfaction
WM2		1.000				
WM3		0.620				
WM4		0.996				
WM5	1.000					
WM6	0.965					
WM8		0.939				
WM9		1.066				
WM10		0.808				
WM11	0.872					
WM12	1.370					
WM14		0.978				
WM15		0.913				
WM16		0.961				
WM17	1.177					
WM18	1.421					
WM19		0.605				
MLLL1			1.000			
MLLL2			0.641			
MLLL3			1.383			
MLLL4			0.894			
MLLL5			1.431			
MLLL6			1.368			
MLLL7			0.773			
MLLL8			0.900			
MLLL9			0.991			
MLLL10			1.623			
MLLL11			1.500			
MLLL12			1.263			
MLLL13			1.647			
MLLL14			0.783			
AUT1				1.000		
AUT2				0.910		
AUT3				0.875		
AUT4				1.050		
AUT5				0.954		
AUT6				0.684		
AUT7				0.733		
AUT8				0.905		
COMP1					1.000	
COMP2					1.504	
COMP3					1.039	
COMP4					1.385	
COMP5					1.012	
COMP6					1.847	
COMP7					0.950	
COMP8					1.052	
REL1						1.000
REL2						0.905
REL3						1.042
REL4						0.821
REL5						0.889
REL6						0.726
REL7						0.931
REL8						0.469
REL9						1.025
REL10						0.999
REL11						1.098
REL12						1.198

*WM* = work motivation. WM1, WM7 and WM13 are the items for amotivation, which are left out because we do not include amotivation in our analysis. MLLL = lifelong learning motivation, AUT = Autonomy, COMP = Competence, REL = Relatedness

**Table 5:** Goodness of fit indices for all variables

	χ^2^ *P*-value	RMSEA	CFI	TLI
AM (autonomous motivation)	106.1 0.052	0.05	0.96	0.94
CM (controlled motivation)	0.092 0.95	0.05	0.95	0.93
Lifelong learning motivation	211 0.00	0.06	0.92	0.82
Autonomy satisfaction	117.9 0.075	0.05	0.96	0.94
Competence satisfaction	46.3 0.102	0.03	0.95	0.92
Relatedness satisfaction	282.2 0.006	0.04	0.98	0.89

## Abbreviations

AM: Autonomous motivation
CFI: Comparative fit index
CM: Controlled motivation
CPD: Continuing professional development
RMSEA: Root mean square error approximation
SDT: Self-determination Theory
TLI: Tucker-Lewis index
