# Correction to: Medical specialists’ basic psychological needs, and motivation for work and lifelong learning: a two-step factor score path analysis

**DOI:** 10.1186/s12909-020-02108-2

**Published:** 2020-06-17

**Authors:** Stéphanie M. E. van der Burgt, Rashmi A. Kusurkar, Janneke A. Wilschut, Sharon L. N. M. Tjin A Tsoi, Gerda Croiset, Saskia M. Peerdeman

**Affiliations:** 1 Amsterdam UMC, Vrije Universiteit Amsterdam, Research in Education, VUmc School of Medical Sciences, de Boelelaan, 1117 Amsterdam, The Netherlands; 2 Department of Epidemiology & Biostatistics, VU University Medical Center Amsterdam, Amsterdam, The Netherlands; 3 PAOFarmacie, The Netherlands Centre for Post-Academic Education in Pharmacy, Amsterdam, The Netherlands; 4 Department of Neurosurgery, VU University Medical Center Amsterdam, Amsterdam, The Netherlands; 5 LEARN! Research Institute for Learning and Education, Faculty of Psychology and Education, VU University Amsterdam, Amsterdam, The Netherlands

**Correction to: BMC Med Educ 19, 339 (2019)**

**https://doi.org/10.1186/s12909-019-1754-0**

In the original publication of this article [1] there are 2 errors related to the location for the quantitive study in the ‘Methods’ section of the article. The incorrect & correct information is published below.

- **Incorrect:** A quantitative study was conducted in an academic hospital (VU University Medical Center in Amsterdam), **a large merged medical center (Noordwestgroep Alkmaar and OLVG Amsterdam**), and two affiliated hospitals (Westfriesgasthuis Hoorn and Rode Kruis Ziekenhuis Beverwijk).
- **Correct:** A quantitative study was conducted in an academic hospital (VU University Medical Center, Amsterdam), **two large merged teaching hospitals (NWZ groep Alkmaar, Spaarnegasthuis Haarlem**) and two affiliated teaching hospitals (Westfriesgasthuis Hoorn and Rode Kruis Ziekenhuis Beverwijk).
